# Service availability and readiness for diabetes and hypertension care among health facilities in Lagos State, Nigeria

**DOI:** 10.1186/s12875-026-03270-0

**Published:** 2026-03-19

**Authors:** Bolanle F. Banigbe, Nafisa Halim, Tobias F. Rinke de Wit, Patricia Elliott, Emmanuella Zamba, Temitope Oke, Ibironke Dada, Gloria P. Gómez-Pérez, Veronika J. Wirtz, Lora L. Sabin

**Affiliations:** 1 Resolve to Save Lives, New York, USA; 2 Department of Global Health, Boston University, Massachusetts, USA; 3 Amsterdam Institute for Global Health and Development, Amsterdam, The Netherlands; 4 PharmAccess, Amsterdam, The Netherlands; 5 Department of Community Health Sciences, Boston University, Massachusetts, USA; 6 Lagos State Health Management Agency, Lagos, Nigeria; 7 PharmAccess Nigeria, Lagos, Nigeria

**Keywords:** Service readiness, Hypertension, Diabetes, Health systems capacity, Service availability, Health insurance

## Abstract

**Background:**

More than 80% of the global premature mortality from noncommunicable diseases (NCDs) occurs in low- and middle-income countries (LMICs). Nigeria, like most LMICs, has limited capacity to respond to diabetes and hypertension. As the Lagos State government accelerates the rollout of its mandatory health insurance, Lagos State Health Scheme (LSHS), the number of individuals with diabetes and hypertension seeking care will increase. This study aimed to determine service availability and service readiness for diabetes and hypertension among health facilities providing primary care for these conditions in Lagos State, and to explore the facility characteristics associated with service readiness.

**Methods:**

We conducted a cross-sectional survey of 84 facilities enrolled in the baseline study of an impact evaluation of the Lagos State Health Scheme. We collected data using relevant modules of the World Health Organization’s Harmonized Health Facility Assessment tool. Service availability was defined as providing diagnosis or treatment for either condition, and service readiness scores were calculated as the proportion of tracer items available and functional at the facility on the survey day. Further, we used a multiple linear regression model to estimate associations between facility characteristics and service readiness.

**Results:**

Service availability for both conditions was high. The mean diabetes and hypertension service readiness scores were 69% and 66%, respectively. The percentage of fully ready healthcare facilities was very low (2.6% for diabetes and 2.5% for hypertension). The staff and guidelines domain received the lowest score for both conditions. There was no association between service readiness and LSHS empanelment status. Providing only outpatient services had a negative association with service readiness for both conditions. Participation in a quality improvement program had a positive association with hypertension service readiness score.

**Conclusion:**

While the mean service readiness scores for diabetes and hypertension were moderately high among sampled health facilities, only a very small percentage were fully service ready. There were critical deficits in service readiness domains that must be addressed to ensure the required inputs for high-quality diabetes and hypertension care is available in the State.

**Supplementary Information:**

The online version contains supplementary material available at 10.1186/s12875-026-03270-0.

## Introduction

Low- and middle-income countries (LMICs) like Nigeria bear a disproportionate burden of the global non-communicable diseases (NCD) mortality with cardiovascular disease (CVD) being the most common cause [1, 2]. The growing burden of risk factors like diabetes, hypertension, air pollution, and unhealthy lifestyles [3–6] suggests that they will become an even more critical health concern in coming years.

The World Health Organization’s (WHO) recommendations for a national NCD response prescribes significant roles for health systems in prevention, education, and treatment centered around primary health care [2]. People living with NCDs require long-term pharmacological and non-pharmacological management delivered in a planned and integrated manner [7–9]. However, health systems capable of delivering this type of care for diabetes and hypertension must have sufficient inputs and functional systems to ensure care continuity and coordination, prioritize care quality, and use information systems to improve care planning and delivery [9–11]. The available evidence on NCD care capacity suggests that health systems in most LMICs face large challenges providing such care [12–15]. In addition to lacking the required inputs and resources to diagnose and investigate NCDS [7, 11], LMIC health systems have an acute, episodic orientation to patient management [10].

In Nigeria, CVD was the fourth leading cause of death in 2021 [16]. It accounted for about 33% of all NCD deaths, while diabetes was responsible for about 5% of NCD-attributable deaths [17]. The prevalence of CVD risk factors is also high and growing; about 30% of adults have hypertension [18], about 5% have diabetes [19], while the average daily salt intake is 16% above WHO recommendations [20]. Considering Nigeria’s large population and high prevalence of CVD risk factors, there is an urgent need for interventions to address the high burden.

Achieving the goal of at least 25% relative reduction in premature NCD mortality by 2025 set by the Nigeria multisectoral action plan (MSAP) [21] requires a robust health system capable of delivering required services. However, little has been published on the capacity of the Nigerian health system to respond to diabetes and hypertension. The national NCD profiles published by the WHO paint a dire picture of the overall national response [22–24]. For instance, between 2017 and 2022, Nigeria reported having no approved national guidelines for managing the four major NCDs. Nigeria also failed to achieve the goal that at least 50% of primary health care facilities offer cardiovascular risk stratification for the management of patients at high risk for heart attack and stroke and have basic medications to manage diabetes and hypertension [13, 25].

Regarding health facility capacity, at the time of this study, only two NCD service readiness surveys were published for Nigeria. They were conducted in two states [26, 27], and their results are not generalizable to other states. First, they focused on public sector facilities, although the private sector provides about a third of primary and secondary care services in Nigeria [28]. Additionally, neither paper explored the facility characteristics associated with service readiness. This highlights a gap in our knowledge regarding service readiness for diabetes and hypertension care in Nigeria and its associated factors. To develop state and local government areas (LGA) specific plans for achieving national NCD control targets, current and context-specific data on health facilities that provide primary care for diabetes and hypertension are critical.

This study aimed to assess the readiness of health facilities providing primary care in Lagos State for diabetes and hypertension services and to explore facility characteristics associated with service readiness.

## Methods

### Design

Ours is a cross-sectional study using the baseline data of an impact evaluation of the Lagos State Health Scheme (LSHS). The impact evaluation (parent study) will utilize a quasi-experimental study design to determine the effect of participation in the LSHS on the quality of care and facility service readiness for diabetes, hypertension, antenatal care, and child curative services as well as business performance among health facilities in Lagos State. Baseline data were collected between October and December 2020.

### Questions and hypotheses

This paper aims to answer the questions: what is the diabetes and hypertension service readiness status among the health facilities? Which facility characteristics are associated with service readiness?

We hypothesized that (1) facilities participating in the LSHS have higher service readiness scores; and (2) facilities that received additional physical resources or program support have higher service readiness scores. We defined receipt of additional support using four variables: (i) participation in any other health insurance scheme (national health insurance scheme (NHIS) only, private health insurance scheme (PHIS only), or both NHIS and PHIS), (ii) receipt of additional funds or equipment from external donors in the 18 months prior(yes/no), (iii) participation in any clinical quality assurance program in the 18 months prior (yes/no), and (iv) participation in the SafeCare program (yes/no).

### Setting

This study took place in Lagos State, Nigeria. Health care services in Lagos are provided by both public and private sectors; the latter has for-profit as well as not-for-profit actors [29]. Overall, only 10% of health facilities in the state are public, which include primary, secondary, and tertiary facilities. The LSHS, established in 2015, is a state-based contributory health insurance program that aims to improve access to high-quality health care services, while reducing out-of-pocket expenditures and catastrophic health spending among Lagosians [30, 31]. Participation is mandatory for residents who lack health insurance coverage from other sources [32].

Health facilities become eligible to participate in the scheme and provide care to enrollees through an empanelment process. This process is voluntary for private facilities, which must express an interest in participation. They are then assessed by the state-owned Health Facility Monitoring and Accreditation Agency (HEFAMAA) and must score at least 60% in an assessment of the adequacy of their physical infrastructure and human resources to be eligible for empanelment^1^. Passing facilities undergo an administrative validation process and orientation with the Lagos State Health Management Agency (LASHMA) before having enrollees assigned. Public facilities also go through this process; however, they are recommended for participation by their managing agency. Upon empanelment in the scheme, facilities receive upfront financial investment from the government to improve their operations. Additionally, all empaneled facilities are required to register for and participate in a quality improvement program, SafeCare [33], to facilitate delivery of high-quality care. These facilities also benefit from periodic quality audits led by the Lagos State Health Management Agency to assess quality of care and provide actionable feedback. While the leadership of non-LSHS facilities may choose to enroll their facilities in SafeCare, they do not have access to upfront cash injection or periodic quality audits.

### Sampling and sample size

Facilities eligible to participate in the LSHS formed the sampling frame for this study. The sampling frame was constructed using data from two sources, the list of all facilities assessed by HEFAMAA for empanelment and the list of empaneled health facilities posted on LASHMA’s website [34]. As of September 2020, HEFAMAA had assessed about 500 facilities, 431 of which scored at least 60%. Of the 431 facilities, 351 were privately-owned, 55 were public primary health centers (PHC), and 25 were public secondary facilities^2^. The required sample size, n, is given as p [1-p]/e^2^, where p is the proportion of primary health centers (25.2%) which had at least one unexpired antihypertensive in an earlier study [35], and e is the standard error (5%). A correction formula for study populations < 10, 000 was applied [36], to yield 84 health facilities in total.

The parent study utilized a two-stage stratified random sampling technique to select facilities. All facilities in the sampling frame were first classified into intervention or comparison group based on empanelment status on September 30, 2020. The intervention group comprised public and private facilities that had passed LASHMA’s assessment process and were empaneled in the scheme by September 30, 2020 (*n* = 187). The comparison group comprised public and private facilities that qualified for LASHMA assessment (*n* = 244), having fulfilled all HEFAMAA requirements, but had not yet applied for empanelment with LASHMA by September 30, 2020. Facilities in each group were then stratified by ownership (public or private), and by level of care (primary or secondary). Twelve facilities were selected randomly for each stratum using a random sample generator in SPSS [37], with equal numbers in each arm.

### Data collection

Data were collected using a facility survey tool (Annex 1) adapted from the diabetes and cardiovascular disease modules of the WHO harmonized health facility assessment (HHFA) tool [38]. The tool was coded on the open data kit (ODK) Collect app v1.29.3 and loaded on Android tablets. A team of trained research associates with medical or social science background collected the data. The survey was administered onsite to the most senior staff member(s) available on the day of the survey at all facilities that reported diagnosing or treating patients with diabetes or hypertension in 2020. The research team also visited service delivery areas, the laboratory, and the pharmacy to observe availability of medications, equipment, and supplies. Data were entered into the ODK app in real time.

### Variables

Service availability was defined as the proportion of facilities that responded “yes” to the question “does this facility provide diagnosis or treatment for diabetes or cardiovascular diseases?“. Service readiness was defined according to the WHO service availability and readiness assessment (SARA) [39, 40] in two ways: a service readiness score, which was the proportion of condition-specific tracer items available and functional on the survey day, and service readiness status. SARA defines the essential inputs needed for service-specific readiness across four domains: (i) trained staff and relevant and up-to-date guidelines; (ii) functioning equipment; (iii) diagnostic capacity; and (iv) essential medicines and commodities.

Following WHO recommendations [40], each facility received a score of 1 point if it reported the availability of an item in the relevant domain. Service readiness status was coded as binary, with 1 = fully ready for diabetes or hypertension care and 0 = not fully ready. A health facility was “ready” if all tracer items were available and functional on the survey day. The service readiness score is a weighted average of all tracer items that were available and functional on the survey day; it ranged between 0 and 100. We also assessed the availability of additional equipment and laboratory tests deemed necessary for optimal diabetes care but not included in the SARA tool e.g. 10 g monofilament [41], although they were not included in the service readiness index to ensure comparability of our results with existing literature.

### Statistical analysis

Continuous variables were summarized using means and standard deviations or medians and interquartile range depending on the skewness of the underlying data, while categorical variables were summarized with frequencies and proportions. For service availability, we calculated the percentage of facilities that reported providing diabetes or CVD services, described components of diabetes and CVD services provided by each facility, and calculated the proportion of facilities offering the full complement of diabetes and cardiovascular disease services as described by the WHO HHFA. We then tested differences in service availability rates by facility characteristics using the chi-squared test or the Fisher’s exact test.

Next, we estimated availability of each item making up service readiness domains using frequencies and proportions, as well as means and standard deviations. Finally, we calculated diabetes and hypertension readiness scores and service readiness status for each facility that reported availability of the service. We then compared service readiness domain and overall readiness scores by facility characteristics using the chi-squared test or Fisher’s exact test. We visually explored variation in service readiness scores across facility categories using grouped box plots.

We developed three sequential linear regression models to assess and isolate the effects of the variables pertinent to the hypotheses regarding diabetes and hypertension service readiness scores: facility characteristics associated with service readiness for each condition, including ownership, service levels, rural-urban location [14, 15, 42–44] (Model 1), receipt of additional support (Model 2) [15, 31, 45, 46], and the full model (Model 3).We estimated a variant of the equation:

$$=\beta_O + \beta_i\mathrm{F}_i + \beta_j\mathrm{i} + \epsilon$$

where $\gamma$= diabetes readiness score or hypertension readiness score, F represents an array of variables related to facility characteristics, and S represents an array of variables related to measures of additional support received by the facility.

To assess model fit, we performed visual diagnostic tests and tested models for multicollinearity using the “mctest” package in R [47] and examined each model’s variance inflation factor (VIF). We adopted a 5.0 cutoff for multicollinearity. For diabetes service readiness, we dropped one of a pair of related explanatory variables that might explain the high VIF. Data were analyzed in R statistical software version 4.0.3 [48]; we employed the “lm” function for multiple linear regression analysis.

## Results

### Facility characteristics

We surveyed 84 clinics across 19 local government areas (LGAs) of Lagos State (Table 1); 51.2% were empaneled in the LSHS. About one-quarter (27.4%) were primary-level facilities, and 66.7% were private health facilities. Most, 90.5%, were located in urban LGAs. More than three-quarters (76.2%) of facilities offered both inpatient and outpatient services. More than half (58.3%) participated in the national health insurance scheme (NHIS), the Federal government’s health insurance scheme, and private health insurance schemes administered by health maintenance organizations. About half (52.4%) of facilities reported having at least one donor-funded program in the prior eighteen months. The most reported donor-funded program was tuberculosis diagnosis and treatment (45.2%), followed by human immunodeficiency virus (HIV) care and treatment (28.6%). Among facilities participating in any donor-funded program, 47.7% reported receiving financial support or clinical equipment from donors. About two-thirds (69%) of facilities had participated in some quality improvement (QI) program in the prior 18 months. More than a third (36.9%) were participating in the SafeCare program [33], while 32.1% participated in other QI programs, including those sponsored by the Lagos State government, partner non-governmental organizations, donors, and internal QI efforts.

**Table 1:** Facility characteristics

Characteristic	*N* = 84
LSHS Status, n (%)	
Empaneled	43 (51.2%)
Not Empaneled	41 (48.8%)
Level of Care, n (%)	
Primary	23 (27.4%)
Secondary	61 (72.6%)
Facility Ownership, n (%)	
Private	56 (66.7%)
Public	28 (33.3%)
LGA^&^ Type, n (%)	
Rural	8 (9.5%)
Urban	76 (90.5%)
Level of Services Available, n (%)	
Inpatient and Outpatient	64 (76.2%)
Outpatient Only	20 (23.8%)
Participates in any other Health Insurance Scheme, n (%)	
Both NHIS^#^ and PHIS^^^	49 (58.3%)
NHIS Only	8 (9.5%)
PHIS Only	6 (7.1%)
Does not participate in any other Health Insurance Scheme	21 (25%)
Type of QI Program, n (%)	
No Quality Improvement Program	26 (31%)
SafeCare QI Program	31 (36.9%)
Other Quality Improvement Program	27 (32.1%)
Participates in Donor Funded Program, n (%)	44 (52.4%)
HIV Care and Treatment Program	24 (28.6%)
Tuberculosis Program	38 (45.2%)
Nutrition Program	5 (6%)
Child Health Program	13 (15.5%)
Maternal Health Program	16 (19%)
Others	3 (3.6%)
Donor funding or equipment support, n (%)	21 (47.7%)
Diagnoses or treats any chronic disease, n (%)	80 (95.2%)

*LSHS- Lagos State Health Scheme

^&^LGA- Local Government Area

^#^NHIS- National Health Insurance Scheme

^^^PHIS- Private Health Insurance Scheme

### Diabetes and hypertension service availability

93% (93%) of facilities reported diagnosing or treating diabetes (Table 2, Supplemental Figure 1). The availability of diabetes service components ranged from 94.4% for providing follow up to DM patients to 98.7% for diagnosing DM (Table S1), Overall, 92.3% of facilities that reported diabetes service availability provided all three diabetes service components (Figure S2).

**Table 2:** Diabetes and cardiovascular service availability by selected facility characteristics

Characteristic		LSHS Status			Level of Care			Facility Ownership		
	Overall, *N* = 84^1^	Empaneled, *N* = 43	Not Empaneled, *N* = 41	*p*-value^2^	Primary, *N* = 23	Secondary, *N* = 61	*p*- value^2^	Private, *N* = 56	Public, *N* = 28	*p*- value^2^
Diagnoses or treats Diabetes, n, (%)	78 (92.9%)	41 (95.3%)	37 (90.2%)	0.4	19 (82.6%)	59 (96.7%)	0.05	54 (96.4%)	24 (85.7%)	0.09
Diagnoses and Manages CVDs, n,(%)	79 (94%)	41 (95.3%)	38 (92.7%)	0.7	20 (87%)	59 (96.7%)	0.12	54 (96.4%)	25 (89.3%)	0.3

^1^n (%),^2^ Fishers exact test

Nearly all (94%, *n* = 79) study facilities reported diagnosing or treating CVDs (Table 2). The availability of the four CVD service components; hypertension, myocardial infarction, congestive heart failure, and stroke, varied widely, from 40.5% for myocardial infarction to 97.5% for hypertension (Table S2, Figure S3). Secondary facilities and private health facilities were more likely to report the availability of each CVD service component.

### Diabetes service readiness

Overall, 14.1% of the facilities had any guidelines for the diagnosis or management of diabetes; none of them had a physical copy of the national guidelines (Table 3). Almost all (97.4%) reported that clinicians consult online diabetes treatment guidelines from other countries, including facilities with copies of facility-developed guidelines. More than half (56.4%) of the health facilities reported having at least one staff trained to diagnose or manage diabetes in the preceding two years. Only 12.8% of the health facilities had both diabetes treatment guidelines and at least one trained staff.

**Table 3:** Diabetes service readiness domain scores

Diabetes Service Readiness Components				LSHS Status				Level of Care					Facility Ownership		
		Overall, *N* = 78^*1*^		Empaneled, *N* = 41		Not Empaneled, *N* = 37		Primary, *N* = 19		Secondary, *N* = 59			Private, *N* = 54		Public, *N* = 24
Has DM guideline, n,(%)		11 (14.1%)		7 (17.1%)		4 (10.8%)		2 (10.5%)		9 (15.3%)			6 (11.1%)		5 (20.8%)
Trained Staff, n,(%)		44 (56.4%)		22 (53.7%)		22 (59.5%)		9 (47.4%)		35 (59.3%)			30 (55.6%)		14 (58.3%)
Both Trained Staff and Guidelines, n,(%)		10 (12.8%)		6 (14.6%)		4 (10.8%)		1 (5.3%)		9 (15.3%)			6 (11.1%)		4 (16.7%)
Guideline and Trained Staff Domain Score, Mean (SD)		35.3 (34.3)		35.4 (35.8)		35.1 (33.1)		28.9 (30.3)		37.3 (35.5)			33.3 (33.6)		39.6 (36.1)
Adult Weighing Scale, n,(%)		74 (94.9%)		38 (92.7%)		36 (97.3%)		17 (89.5%)		57 (96.6%)			54 (100%)		20 (83.3%)^
Measuring Tape, n,(%)		70 (89.7%)		40 (97.6%)		30 (81.1%)^**^		16 (84.2%)		54 (91.5%)			49 (90.7%)		21 (87.5%)
Blood Pressure Apparatus, n,(%)		77 (98.7%)		40 (97.6%)		37 (100%)		19 (100%)		58 (98.3%)			54 (100%)		23 (95.8%)
Stethoscope, n,(%)		77 (98.7%)		40 (97.6%)		37 (100%)		19 (100%)		58 (98.3%)			53 (98.1%)		24 (100%)
Glucometer, n,(%)		65 (83.3%)		35 (85.4%)		30 (81.1%)		14 (73.7%)		51 (86.4%)			47 (87%)		18 (75%)
Unexpired Glucometer Test Strip, n,(%)		65 (83.3%)		35 (85.4%)		30 (81.1%)		14 (73.7%)		51 (86.4%)			47 (87%)		18 (75%)
10 g Monofilaments, n,(%)^#^		7 (9%)		5 (12.2%)		2 (5.4%)		1 (5.3%)		6 (10.2%)			5 (9.3%)		2 (8.3%)
BMI chart/Wheel, n,(%)^#^		9 (11.5%)		6 (14.6%)		3 (8.1%)		2 (10.5%)		7 (11.9%)			7 (13.0%)		2 (8.3%)
Ophthalmoscope, n,(%)^#^		26 (33.3%)		15 (36.6%)		11 (29.7%)		4 (21.1%)		22 (37.3%)			21 (38.9%)		5 (20.8%)
Stadiometer/Height board, n,(%)^#^		54 (69.2%)		27 (65.9%)		27 (73%)		12 (63.2%)		42 (71.2%)			39 (72.2%)		15 (62.5%)
Patella Hammer, n,(%)^#^		42 (53.8%)		23 (56.1%)		19 (51.4%)		4 (21.1%)		38 (64.4%)^$^			36 (66.7%)		6 (25%)^$^
Equipment Domain Score, Mean (SD)		91.5 (16.5)		92.7 (17.5)		90.1 (15.4)		86.8 (18.9)		92.9 (15.5)			93.8 (13.8)		86.1 (20.7)
Metformin, n,(%)		68 (87.2%)		37 (90.2%)		31 (83.8%)		15 (78.9%)		53 (89.8%)			47 (87.0%)		21 (87.5%)
Insulin Injection, n,(%)		51 (65.4%)		27 (65.9%)		24 (64.9%)		3 (15.8%)		48 (81.4%)^$^			43 (79.6%)		8 (33.3%)^$^
Gliclazide, n,(%)		28 (35.9%)		15 (36.6%)		13 (35.1%)		5 (26.3%)		23 (39.0%)			20 (37.0%)		8 (33.3%)
Glibenclamide, n,(%)		58 (74.4%)		30 (73.2%)		28 (75.7%)		12 (63.2%)		46 (78.0%)			42 (77.8%)		16 (66.7%)
50% Glucose Injection, n,(%)		43 (55.1%)		21 (51.2%)		22 (59.5%)		4 (21.1%)		39 (66.1%)^$^			33 (61.1%)		10 (41.7%)
Intermediate Acting Insulin, n,(%)#		32 (41%)		19 (46.3%)		13 (35.1%)		0 (0%)		32 (54.2%)^$^			27 (50%)		5 (20.8%)^**^
Medications Domain Score, Mean (SD)		63.6 (30)		63.4 (27.9)		63.8 (32.6)		41.1 (26.2)		70.8 (27.6)^$^			68.5 (29.7)		52.5 (28.2)^++^
Urine Ketone, n,(%)		63 (80.8%)		33 (80.5%)		30. (81.1%)		12 (63.2%)		51 (86.4%)^*^			46 (85.2%)		17 (70.8%)
Urine Proteins, n,(%)		71 (91%)		38 (92.7%)		33 (89.2%)		18 (94.7%)		53 (89.8%)			48 (88.9%)		23 (95.8%)
Any Glucose Testing, n,(%)		66 (84.6%)		36 (87.8%)		30 (81.1%)		15 (78.9%)		51 (86.4%)			47 (87%)		19 (79.2%)
HbA1C, n,(%)		23 (29.5%)		14 (34.1%)		9 (24.3%)		2 (10.5%)		21 (35.6%)^*^			18 (33.3%)		5 (20.8%)
Diagnostics Domain Score, Median (IQR)		100 (66.7,100)		100 (83.3,100)		100 (66.7,100)		100 (66.7,100)		100 (100,100)^&&^			100 (100,100)		100 (66.7,100)
Diabetes Service Readiness Score, Mean (SD)		68.9 (16.9)		69.6 (15.2)		68.2 (18.9)		58.9 (15.6)		72.2 (16.2)^&^			70.7 (16.7)		65.0 (17.1)^&&^
Ready for Diabetes Services, n,(%)		2 (2.6%)		1 (2.4%)		1 (2.7%)		0 (0%)		2 (3.4%)			1 (1.9%)		1 (4.2%)

Mean (SD) 2Fisher’s exact test; Pearson’s Chi-squared test; Wilcoxon rank sum test; two-sample t-test

** *p* = 0.02

*P = 0.04

^1^n (%)

^#^ Not included in the diabetes service readiness score elements

^&^*P* = 0.001

^$^ *p* < 0.001

^, *p* = 0.007

^++^ *p* = 0.01

^&&^ 0.06

Equipment availability ranged from 9% for 10 g monofilaments to 98.7% for stethoscopes and blood pressure measuring devices. More than three-quarters (83.3%) of facilities had a functioning glucometer, and every facility with a functioning glucometer had at least one unexpired glucometer test strip. Metformin was available at 87.2% of facilities; about two-thirds (65.4%) had insulin. Both regular and intermediate acting insulin were more likely to be available at secondary and private facilities. The mean overall medication domain score was 63.6% (SD: 30). The medication domain score was significantly higher among secondary facilities (*p* < 0.001) and private health facilities (*p* = 0.03).

Most facilities, 84.6%, had onsite blood glucose testing capacity, while about a third, 29.5%, had onsite capacity for glycated hemoglobin (HbA1C) testing; secondary facilities were more likely to have onsite capacity for HbA1C (*p* = 0.04). The mean diabetes readiness score was 68.9% (SD: 16.9). Secondary facilities were more likely to receive a higher diabetes service readiness score than primary facilities (*p* = 0.001).

**Fig. 1:**
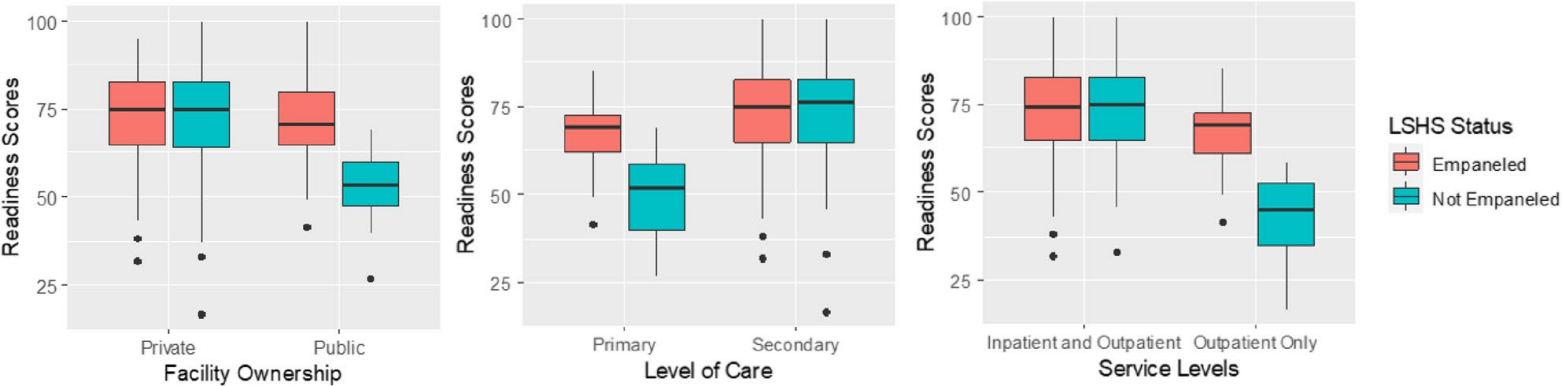
Box plots showing the variation in mean diabetes readiness scores by facility characteristics

As shown in Fig. 1, the mean diabetes service readiness score was similar among private facilities and secondary facilities, and those providing both inpatient and outpatient services regardless of LSHS participation status. In contrast, primary facilities and public facilities and those providing inpatient services only participating in the LSHS had higher diabetes service readiness scores than their non-LSHS counterparts.

Only two, 2.6%, of facilities were fully ready to deliver diabetes services (Table 3); both fully ready facilities were secondary level facilities located in urban LGAs.

### Determinants of diabetes service readiness

Table 4 presents the results of three linear regression models developed to assess the relationship between diabetes service readiness scores and health facility characteristics (Model 1), receipt of additional support (Model 2), and the full model (Model 3).

**Table 4:** Linear regression models investigating factors associated with diabetes service readiness scores

Characteristic	Model 1			Model 2			Model 3		
	Beta	95% CI^1^	p-value	Beta	95% CI^1^	p-value	Beta	95% CI^1^	p-value
Service Level									
Inpatient and Outpatient	Ref	Ref					Ref	Ref	
Outpatient Only	−25	−36, −14	<0.001				−21	−33, −8.5	0.001
Facility Ownership									
Private	Ref	Ref					Ref	Ref	
Public	16	4, 28	0.01				18	2.6,34	0.02
LSHS Status									
Empaneled	Ref	Ref					Ref	Ref	
Not Empaneled	3.5	−4.6, 12	0.4				3.9	−4.4, 12	0.4
LGA Type									
Rural	Ref	Ref					Ref	Ref	
Urban	0.09	−12, 13	>0.9				3.7	−9.7, 17	0.6
Facility Ownership * LSHS Status									
Public * Not Empaneled	−12	−36, −0.5	0.009				−19	−35, −2.1	0.03
Participates in any other Health Insurance Scheme									
Both NHIS and PHIS				Ref	Ref		Ref	Ref	
NHIS Only				−6.4	−19, 6.8	0.3	−8.6	−23, 5.7	0.2
No				−12	−21, −2.6	0.01	−7.8	−19, 3.1	0.2
PHIS Only				−17	−31, −3.7	0.01	−12	−27, 2.8	0.1
Type of QI Program									
No QI Program				Ref	Ref		Ref	Ref	
Other QI Program				6.4	−2.9, 16	0.2	5.4	−3.7, 14	0.2
SafeCare QI Program				12	2.7, 20	0.01	8.2	−0.5, 17	0.06
Donor funding or equipment support									
No				Ref	Ref		Ref	Ref	
Yes				2.2	−6.6, 11	0.6	0.5	−9.1, 10	>0.9

^1^CI = Confidence Interval

Two facility characteristics—providing outpatient services only and being a non-LSHS public facility—were associated with a lower diabetes readiness score. None of the variables measuring receipt of additional physical resources was significantly associated with diabetes service readiness score in the full model. In Model 2, participation in the SafeCare QI program was associated with a higher diabetes readiness score (β: 12, 95%CI: 2.7–20), however, it was not statistically significant in the full model after controlling for other facility characteristics.

### Hypertension service readiness

Overall, only 10.1% of the facilities had any guidelines for diagnosis or management of hypertension (Table 5). About 13% (12.7%) of facilities reported that clinicians consult online hypertension treatment guidelines developed for other countries. Only 5.1% had a CVD risk assessment tool available on the day of the visit. About half (49.4%) of the health facilities reported having at least one staff trained to diagnose and treat hypertension in the preceding two years. Only 6.3% of the health facilities had hypertension treatment guidelines and at least one trained staff.

**Table 5:** Hypertension service readiness domain scores

Characteristic		LSHS Status		Level of Care		Facility Ownership	
	Overall, *N* = 79^*1*^	Empaneled, *N* = 41	Not Empaneled, *N* = 38	Primary, *N* = 20	Secondary, *N* = 59	Private, *N* = 54	Public, *N* = 25
Has Hypertension Guideline, n, (%)	8 (10.1%)	4 (9.8%)	4 (11%)	1 (5%)	7 (11.9%)	7 (13%)	1 (4%)
CVD Job Aids or Treatment Algorithms Available, n, (%)	3 (3.8%)	1 (2.4%)	2 (5.3%)	0 (0%)	3 (5.1%)	2 (3.7%)	1 (4%)
CVD Risk Assessment Tool Available, n, (%)	4 (5.1%)	3 (7.3%)	1 (2.6%)	0 (0%)	4 (6.8%)	2 (3.7%)	2 (8%)
Trained Staff, n, (%)	39 (49.4%)	19 (46.3%)	20 (52.6%)	9 (45%)	30 (50.8%)	26 (48.1%)	13 (52%)
Has both Guideline and Trained Staff, n, (%)	5 (6.3%)	3 (7.3%)	2 (5.3%)	1 (5%)	4 (6.8%)	4 (7.4%)	1 (4%)
Guideline and Trained Staff Domain Score, Mean (SD)	29.8 (30.5)	28 (32)	32 (29)	25 (30.3)	31.4 (30.7)	30.6 (31.3)	28 (29.2)
Adult Weighing Scale, n, (%)	75 (94.9%)	38 (92.6%)	37 (97.3%)	18 (90%)	57 (96.6%)	54 (100%)	21 (84%)^^^
Blood Pressure Apparatus, n, (%)	77 (97.5%)	40 (97.6%)	37 (97.3%)	19 (95%)	58 (98.3%)	54 (100%)	23 (92%)
Stethoscope, n, (%)	77 (97.5%)	40 (97.6%)	37 (97.3%)	19 (95%)	58 (98.3%)	53 (98.1%)	24 (96%)
Oxygen, n, (%)	54 (68.4%)	27 (65.8%)	27 (71.1%)	10 (50%)	44 (74.6%)^*^	40 (74.1%)	14 (56%)
Equipment Domain Score, Median (IQR)	100 (75,100)	100 (75,100)	100 (75,100)	100 (75,100)	100 (75,100)^**^	100 (75,100)	100 (75,100)^&^
Calcium Channel Blockers, n, (%)	73 (92.4%)	39 (95.1%)	34 (89.5%)	17 (85%)	56 (94.9%)	50 (92.6%)	23 (92%)
Thiazide Diuretics, n, (%)	61 (77.2%)	33 (80%)	28 (74%)	12 (60.0%)	49 (83.1%)^&&^	43 (79.6%)	18 (72%)
Angiotensin Converting Enzymes Inhibitor, n, (%)	53 (67.1%)	28 (68.3%)	25 (65.8%)	12 (60%)	41 (69.5%)	36 (66.7%)	17 (68%)
Beta Blocker, n, (%)	53 (67.1%)	31 (75.6%)	22 (57.9%)	6 (30%)	47. (79.7%)^$^	41 (75.9%)	12 (48%)^++^
Acetyl Salicylic Acid, n, (%)	70 (88.6%)	36 (87.8%)	34 (89.5%)	16 (80%)	54 (91.5%)	48 (88.9%)	22 (88%)
Medications Domain Score, Mean (SD)	78.5 (26.4)	81 (24)	75 (28)	63 (28.5)	83.7 (23.6)^$^	80.7 (25.5)	73.6 (28.1)
Hypertension Service Readiness Score, Mean (SD)	66.0 (16)	66 (15)	66 (17)	56.8 (18.9)	69 (13.6)^^^^	68.1 (14.2)	61.2 (18.4)
Ready for Hypertension Services, n, (%)	2 (2.5%)	1 (2.4%)	1 (2.6%)	0 (0%)	2 (3.4%)	2 (3.7%)	0 (0%)

Pearson’s Chi-squared test; Wilcoxon rank sum test

*P = 0.04

** p = 0.02

^1^ n (%); Mean (SD)

^2^ Fisher’s exact test

^&^P = 0.002

^$^p < 0.001

^p = 0.008

^++^p = 0.01

^&&^ 0.06

^^^^p = 0.03

Equipment availability ranged from 94.9% for weighing scales to 97% for stethoscopes and blood pressure measuring apparatus. A little more than two-thirds (68.4%) of facilities could provide oxygen in the outpatient department on the survey day; secondary health facilities were more likely to report oxygen availability (*p* = 0.04) than their primary level counterparts. The overall median score for the equipment domain was 100% (IQR: 75%- 100%).

Calcium channel blockers were available at 92.4% of facilities, 77.2% had a thiazide diuretic while 67.1%, had angiotensin-converting enzyme (ACE) inhibitors available. Beta-blockers were available at 67.1% of facilities; secondary health facilities (*p* < 0.001) and private facilities (*p* = 0.01) were more likely to have beta-blockers. The overall mean medication domain score was 79% (SD: 26); secondary health facilities received a significantly higher medication domain score compared to the primary health facilities (*p* < 0.001). The mean hypertension service readiness score was 66% (SD: 16). Secondary health facilities achieved a significantly higher hypertension service readiness score than primary health facilities (*p* = 0.03).

**Fig. 2:**
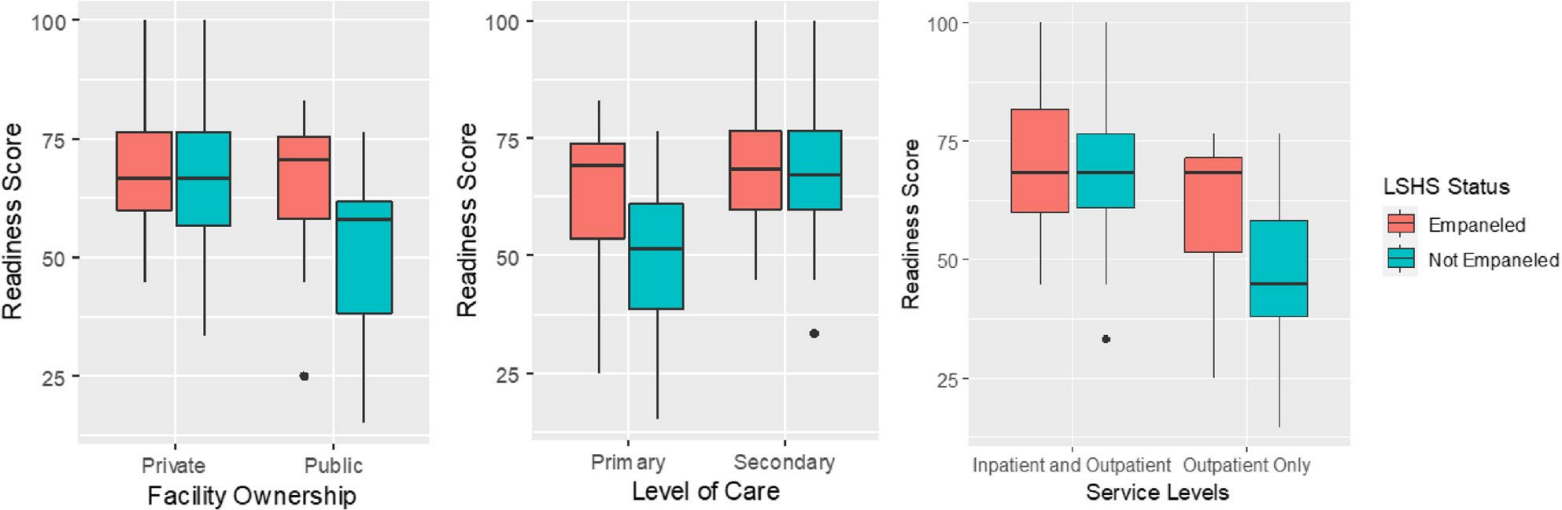
Box plots showing the variation in mean hypertension readiness scores by facility characteristics

As shown in Fig. 2, the mean hypertension service readiness score was similar among private facilities, secondary facilities, and those providing both inpatient and outpatient services regardless of their LSHS participation status. In contrast, primary facilities, public facilities and those providing inpatient services participating in the LSHS had higher hypertension service readiness scores than their non-LSHS counterparts.

Only 2.5% (*n* = 2) of facilities were fully ready to deliver hypertension services, one of them was empaneled in the LSHS. Both facilities were private, secondary facilities located in urban LGAs.

### Determinants of hypertension service readiness

Table 6 presents the results of three linear regression models developed to assess the relationship between hypertension service readiness scores and health facility characteristics (Model 1), receipt of additional support (Model 2), and the full Model (Model 3). Providing outpatient services only was associated with a lower hypertension readiness score (: −16%, 95%CI: −28 -to −4.1) in the full model, while participation in the SafeCare QI program was significantly associated with a higher hypertension service readiness score (β: 10%, 95%CI: 1.4–19).

**Table 6:** Linear regression models investigating factors associated with hypertension service readiness scores

Characteristic	Model 1			Model 2			Full Model		
	Beta	95% CI^1^	*p*- value	Beta	95% CI^1^	*p*-value	Beta	95% CI^1^	*p*-value
Service Level									
Inpatient and Outpatient	Ref	Ref					Ref	Ref	
Outpatient Only	−20	−30, −9	< 0.001				−16	−28, −4.1	0.009
Facility Ownership									
Private	Ref	Ref					Ref	Ref	
Public	4.6	−5.5, 15	0.4				3.7	−10, 17	0.6
LSHS Status									
Empaneled	Ref	Ref					Ref	Ref	
Not Empaneled	−0.7	−7.5, 6.2	0.8				−1.1	−6.0, 8.3	0.8
LGA Type									
Rural	Ref	Ref					Ref	Ref	
Urban	−1.4	−14, 11	0.8				1.0	−12, 14	0.9
Participates in any other Health Insurance Scheme									
Both NHIS and PHIS				Ref	Ref		Ref	Ref	
NHIS Only				−2.6	−15, 10	0.7	−0.7	−15, 13	> 0.9
No				−8.1	−17, 0.5	0.06	−3.7	−14, 6.9	0.5
PHIS Only				−9.7	−23, 3.4	0.14	−1.1	−15, 13	0.9
Type of QI Program									
No QI Program				Ref	Ref		Ref	Ref	
Other QI Program				8.9	0.2, 18	0.05	6.9	−2.0, 16	0.12
SafeCare QI Program				12	3.6, 21	0.006	10	1.4, 19	0.02
Donor funding or equipment support									
No				Ref	Ref		Ref	Ref	
Yes				−0.7	−9.0, 7.7	0.9	0.9	−8.3, 10	0.9

^1^CI = Confidence Interval

## Discussion

Given the knowledge gap about service readiness of healthcare facilities providing diabetes and hypertension care in Lagos State, Nigeria, this study adds important evidence decision makers can use to optimize services for both conditions.

### Service availability

Diabetes service availability among the facilities in this study was higher than the reported21% to 59% [14, 15, 42, 49] in existing literature. Most facilities, 97.5%, in this study reported hypertension service availability, consistent with the findings of a similar study in Abuja, Nigeria, where 97% of primary health centers (PHCs) reported hypertension service availability [26]. None of the examined facility characteristics were associated with service availability for either condition, in contrast to findings in some earlier studies where services were more available at higher level facilities, the private sector, and in urban areas [15, 42, 46]. A potential explanation for this difference could be that Lagos is mostly urban, and this was not a nationally representative sample of health facilities.

Almost all (92%) facilities with diabetes services available provided the full complement of diabetes services, which was higher than the 70% reported from a nationally representative sample in Bangladesh [15, 43]. Unsurprisingly, only 36% of facilities, mostly secondary or private, provided all four CVD services since these require specialized equipment and highly trained staff. This does not represent all CVD services available in Lagos, as we excluded tertiary facilities from the study. However, this finding can help the State Ministry of Health map service availability and plan new service development to maximize geographical access to specialized CVD services.

### Diabetes and hypertension service readiness were moderately high

The overall mean diabetes service readiness score, the proportion of condition-specific tracer items available and functional on the survey day was moderately high at 68.9% and higher than reported by studies in other LMIC settings, which ranged from 26.4% to 49% [15, 42, 43, 46, 49]. Similarly, the overall hypertension service readiness score of 66% was higher than the 18.8% to 62.7% reported in the literature [15, 27, 42, 46, 49, 50]. This finding of moderately high service readiness scores is unsurprising since the facilities in this sample had already successfully completed an assessment process through HEFAMAA to qualify for participation in the LSHS. Passing the HEFAMAA assessment, however, is insufficient to predict service readiness for diabetes or hypertension, as shown by the proportion of facilities full ready to provide services for either condition. This is because the HEFAMAA did not assess the availability of all diabetes or hypertension service readiness elements, especially treatment guidelines or trained staff^3^, which was the worst performing domain for both conditions. LASHMA will therefore need to provide additional guidance to facilities participating in the LSHS to improve their diabetes and hypertension service readiness. Only 2.6% and 2.5% of the facilities were fully ready to provide diabetes and hypertension services, respectively. This is consistent with existing studies that reported these outcomes where less than 1% of facilities were fully ready [15, 42, 51, 52]. Recent systematic review evidence from 8 LMICs show that majority of health facilities are still not ready to provide services for cardiovascular diseases, despite the recent focus on universal health coverage [52].

Surprisingly, there was no association between service readiness scores and facility characteristics such ownership and level of care, unlike some studies that found private facilities were more likely to score higher [43, 53]. However, providing outpatient services only and being a non-LSHS public facility were associated with a lower diabetes readiness score. Similarly, there was no rural-urban difference in scores, although some surveys reported this [43, 46]; however, a study from Enugu, Nigeria, found no difference in NCD readiness scores between rural and urban PHCs [27]. Importantly, only 5% of the facilities in this study were in rural areas.

### Poor availability of treatment guidelines

The poor availability of hypertension treatment guidelines was similar to the 13% reported among PHCs in Abuja, Nigeria [26]. Although some authors found that higher level and private facilities were more likely to have treatment guidelines [43, 49, 51], there was no such relationship in this study. Surprisingly, guidelines were equally unavailable at LSHS facilities, suggesting gaps in the dissemination or adoption of the Lagos State standard treatment guidelines. The fact that 97% of clinics reported that their clinicians consult online diabetes treatment guidelines developed for other countries suggests a clear need for national guidelines. This finding also has implications for how future SARA assessments determine guideline availability, at least in settings with internet access. During facility assessments it may be helpful to go beyond asking for a physical copy of a guideline and ask about the use of electronic guidelines approved by the relevant national health authorities. Although few facility staff could name the websites their clinicians consulted, they consulted a wide range of sources, which may confuse patient management. The generally low availability of trained staff and finding of no difference in their availability by facility characteristics suggests there are limited opportunities for training on diabetes and hypertension management in this environment. “Since this study was conducted, the management of LSHS has disseminated both physical and digital copies of the standard treatment guidelines to participating facilities. It has also established capacity building mechanisms to train healthcare workers in participating facilities to deliver components of the benefit packages, including diabetes and hypertension care. These actions, in addition to periodic quality audits instituted as part of the insurance framework, should have contributed to higher service readiness scores among the participating facilities.”

### Good availability of equipment

Consistent with existing evidence [15, 45, 53], the availability of basic equipment for both conditions was high overall, above 80%, except for oxygen, which was available at about two-thirds of facilities providing cardiovascular services. Few existing studies assessed the availability of oxygen, and they reported lower availability than in our study [15, 44]. Private facilities and secondary level facilities had higher mean equipment scores for hypertension, similar to some earlier findings [53]. Unlike some studies that found higher diabetes equipment availability among private facilities and those at higher levels of care [44, 49, 54], this study found no such difference. The availability of unexpired strips at all facilities with a glucometer was also different than findings from other settings; for instance, a national survey from Bangladesh [43] found that only 32.2% of facilities with a glucometer had test strips.

Additional equipment needed for patients with diabetes was not as readily available as basic equipment; availability ranged from 69.2% for stadiometers to 9% for 10 g monofilaments. Only a few previous studies assessed the availability of additional equipment. For instance, only one study from Uganda assessed the availability of 10 g monofilaments, and they were available at only 3.8% of the facilities surveyed [54]. Monofilaments are important for early diagnosis of foot and eye complications in a primary care setting, and their absence can lower the effectiveness of primary care services to prevent complications like lower limb amputations. Although onsite glucose capacity testing was high at 85%, the non-universal availability of glucose testing in facilities providing DM care is a critical gap to address given the centrality of glucose monitoring to managing diabetes. Only about a third of facilities had the capacity for glycated hemoglobin testing, much higher than the findings of a Ugandan study [54], where only 9.4% had this capacity. However, very few existing surveys assessed the capacity for glycated hemoglobin testing.

### Moderate availability of medication

The medication availability score for diabetes was moderately high (63.6%) and fell in the middle of the range (11% −91.6%) for scores from past LMIC studies [15, 42, 46]. Consistent with some studies from low resource settings [49], diabetes medication availability was highest at private and secondary health facilities in this study. For hypertension, the overall medication availability score, 78%, was higher than scores reported in the literature, which varied widely from 5.4% to 67.6% [15, 26, 27, 42, 46]. Anti-hypertensive medication availability was higher among secondary facilities, consistent with existing literature showing higher-level facilities had more anti-hypertensive medications [49, 51]. However, private facilities in this study were not more likely to have anti-hypertensive medications than their public counterparts, contrary to some earlier findings in the literature [51, 55]. Overall insulin availability (65.4%) was lower than the 79% and 89% reported in a 2021 survey from Ethiopia and Tanzania, respectively [56], but higher than the availability reported by most other studies, which ranged from1%- 44% [14, 15, 42, 55–57]. Consistent with previous findings in similar settings, private and secondary facilities were more likely to have insulin [42, 46, 49]. While metformin availability was high, the lower availability of glibenclamide and gliclazide may mean that patients not doing well on metformin have access to fewer alternatives in health facilities. Accessing medications within health facilities is essential because they tend to be more affordable and are usually quality assured [58]. The WHO HHFA assesses the availability of at least one unexpired dose of each medicine on the survey day without consideration for adequate doses to manage the number of patients receiving care for either condition at the facility. This data element is insufficient to inform strategies to strengthen reliable medicine availability for diabetes and hypertension and should be reviewed. An indicator that considers adequate medicine availability for the number of patients receiving treatment for diabetes or hypertension at the facility will be more informative. The finding that PHCs had fewer drugs than secondary facilities points to the need to strengthen this level of care for diabetes and hypertension readiness. Uncomplicated diabetes and hypertension are primary care amenable conditions, being able to receive treatment closer to home can promote retention in care, and it is cheaper for the health system overall to manage these conditions at the lower level of care.

### Participation in quality improvement programs

Participation in the SafeCare quality improvement (QI) program was significantly associated with hypertension service readiness, although participation in other QI programs was not. This is contrary to the findings of previous studies, which did not identify associations between QI activity and hypertension service readiness [49, 55]. With respect to diabetes service readiness, although not significant in the full model, participation in the SafeCare QI program was associated with better scores in model 2. In a stratified analysis, the association between service readiness for the two conditions and SafeCare level was stronger among facilities at levels two and three, compared to those at SafeCare level one. This indicates that the SafeCare program may be an important way to introduce facility-level systems strengthening activities for diabetes and hypertension care. With longer SafeCare interventions, the above associations could become more significant.

### Receipt of donor funding and technical assistance

Receipt of external donor funding was not associated with service readiness scores for either condition. A possible explanation could be that these programs focus on infectious and maternal and child health conditions, and do not address the main gaps in NCD service readiness especially treatment guidelines, staff training, and medicines. Donor funded programs strengthen the overall health system, through information systems improvement or pharmaceutical supply chain strengthening, for instance, which can positively impact the quality of NCD services. However, their efforts on health workforce training and treatment guideline development tend to be disease focused [59, 60]. One notable exception is HIV/NCD integration projects which train healthcare workers to manage hypertension and diabetes, and support treatment guideline development. However, many of these pilots fall short on sustainable medicine access which prevents implementation at scale [61]. Therefore, a targeted strategy will be needed to leverage the available donor support for diabetes and hypertension.

### Strengths and limitations

This study is the first to assess diabetes and hypertension service readiness among health facilities in Lagos State. It therefore fills a critical gap in our understanding of NCD service readiness in this part of Nigeria. Additionally, this study collected data from many facilities, including ones that vary by ownership and level of care, making the findings more generalizable to many facilities in Lagos State.

This study also has some limitations. The service readiness component assessed whether at least one staff was trained; this is likely insufficient to improve patient care in high-volume facilities, with many clinicians treating patients with diabetes or hypertension. It also did not account for individual clinicians’ self-development courses. The question also assumes that health facilities keep a good record of staff training, hence, the number of trained staff may be different in real life. Secondly, facilities were eligible to participate in this study if they had completed an initial HEFAMAA assessment process; it is possible such facilities were systematically different from facilities that did not present themselves for the assessment, thereby limiting generalizability of our findings.

## Conclusion

In summary, diabetes and hypertension service availability was high and service readiness was moderately high among this group of private and public facilities in Lagos State, however, very few facilities were fully ready to deliver either service. This aligns with recent evidence from 8 LMICs that shows majority of health facilities are still not ready to provide services for cardiovascular diseases, despite the recent focus on universal health coverage [52]. This is despite a recognition that hypertension and diabetes control are viable pathways to achieving the sustainable development goals, and a generally positive trend on UHC index performance among countries [62]. Primary level facilities performing worse than their secondary counterparts stands in contrast to WHO’s recommendation to center national NCD response around primary health care. This low investment is likely contributory factor to the low diabetes and hypertension control rates recorded in most LMICs. Diabetes and hypertension are primary care amenable NCDs, a deliberate investment in PHCs as recommended by the WHO HEARTS technical package, especially by improving access to reliable and affordable medicine supply, training PHC staff, and equipping them with the needed supplies is still an urgent need.

## Supplementary Information

Supplementary Material 1

Supplementary Material 2

Supplementary Material 3

## Data Availability

The datasets used and/or analyzed during the current study are available from the corresponding author on reasonable request.

## Footnotes

1 Personal Communication with HEFAMAA and PharmAccess Foundation Staff.

2 Internal HEFAMAA monitoring and evaluation data.

3 HEFAMAA Facility Inspection Tool for PHCs.
