# Supplementary material for: Service availability and readiness for diabetes and hypertension care among health facilities in Lagos State, Nigeria
Source: BMC Prim Care. 2026 Mar 19;27:165. doi: 10.1186/s12875-026-03270-0 (PMC13122977; doi:10.1186/s12875-026-03270-0)
Supplement: Supplementary file 2 — Supplementary Material 2 [file 12875_2026_3270_MOESM2_ESM.docx]

Supplemental Figures

Supplemental Figure 1: Categorization of health facilities with diabetes or hypertension services available in the sample

Supplemental Figure 2: Diabetes service availability and readiness cascade

Supplemental Figure 3: Hypertension service availability and readiness cascade
