# Supplementary material for: Service availability and readiness for diabetes and hypertension care among health facilities in Lagos State, Nigeria
Source: BMC Prim Care. 2026 Mar 19;27:165. doi: 10.1186/s12875-026-03270-0 (PMC13122977; doi:10.1186/s12875-026-03270-0)
Supplement: Supplementary file 3 — Supplementary Material 3 [file 12875_2026_3270_MOESM3_ESM.docx]

| **Table S1: Diabetes Service Component Availability by Selected Facility Characteristics** | | | | | | | |
| --- | --- | --- | --- | --- | --- | --- | --- |
| Diabetes Service Component |  | LSHS Status | | Level of Care | | Facility Ownership | |
|  | Overall, N = 78^1^ | Empaneled, N = 41 | Not Empaneled, N = 37 | Primary, N = 19 | Secondary, N = 59 | Private, N = 54 | Public, N = 24 |
| Diagnoses Diabetes, n, (%) | 77  (98.7%) | 41  (100%) | 36  (97.3%) | 19  (100%) | 58  (98.3%) | 53 (98.1%) | 24  (100%) |
| Prescribes Treatment for Diabetes, n, (%) | 76  (97.4%) | 41  (100%) | 35  (94.6%) | 19  (100%) | 57  (96.6%) | 52 (96.3%) | 24  (100%) |
| Follow up Care for Diabetic Patients, n, (%) | 74  (94.9%) | 40  (97.6%) | 34  (91.9%) | 18  (94.7%) | 56  (94.9%) | 51 (94.4%) | 23 (95.8%) |
| All three Diabetes Services, n, (%) | 72  (92.3%) | 40  (97.6%) | 32  (86.5%) | 18  (94.7%) | 54  (91.5%) | 49 (90.7%) | 23 (95.8%) |
| ^1^n (%),^2^ Chi-square test. No significant difference in diabetes service component availability by selected facility characteristics | | | | | | | |

Supplementary Table 1: Diabetes Service Component Availability by Selected Facility Characteristics

| **Table S1: Diabetes Service Component Availability by Selected Facility Characteristics** |
| --- |

Supplementary Table 2: Availability of Cardiovascular Disease Care Components

| **Table S2: Availability of Cardiovascular Disease Care Components** | | | | | | | | | | |
| --- | --- | --- | --- | --- | --- | --- | --- | --- | --- | --- |
| **Characteristic** | **LSHS Status** | | | | **Level of Care** | | | **Facility Ownership** | | |
|  | **Overall, N = 79***^1^* | **Empaneled, N = 41** | **Not Empaneled, N = 38** | **p-value***^2^* | **Primary, N = 20** | **Secondary, N = 59** | **p-value***^2^* | **Private, N = 54** | **Public, N = 25** | **p-value***^2^* |
| Diagnoses and Manages Hypertension, n,(%) | | | | >0.9 |  | | 0.4 |  | | 0.5 |
| Diagnosis, treatment and follow up | 77 (97.5%) | 40  (97.6%) | 37  (97.4%) |  | 19  (95%) | 58  (98.3%) |  | 53 (98.1%) | 24  (96%) |  |
| Follow up Only | 2  (2.5%) | 1  (2.4%) | 1  (2.6%) |  | 1  (5%) | 1  (1.7%) |  | 1  (1.9%) | 1  (4%) |  |
| Diagnoses and Manages Myocardial Infarction, n,(%) | | | | >0.9 |  | | **<0.001** |  | | **0.01** |
| Diagnosis, treatment and follow up | 32 (40.5%) | 17  (41.5%) | 15  (39.5%) |  | 0  (0%) | 32  (54.2%) |  | 27  (50%) | 5 (20%) |  |
| Follow up Only | 2  (2.5%) | 1  (2.4%) | 1  (2.6%) |  | 0  (0%) | 2  (3.4%) |  | 2  (3.7%) | 0  (0%) |  |
| No Service/Refer Suspected Case | 45  (57%) | 23  (56.1%) | 22  (57.9%) |  | 20  (100%) | 25  (42.4%) |  | 25 (46.3%) | 20  (80%) |  |
| Diagnoses and Manages Congestive Heart Failure, n,(%) | | | | >0.9 |  | | **<0.001** |  | | **<0.001** |
| Diagnosis, treatment and follow up | 44  (55.7%) | 23  (56.1%) | 21  (55.3%) |  | 0  (0%) | 44  (74.6%) |  | 38  (70.4%) | 6 (24%) |  |
| Follow up Only | 2  (2.5%) | 1  (2.4%) | 1  (2.6%) |  | 0  (0%) | 2  (3.4%) |  | 2  (3.7%) | 0  (0%) |  |
| No Service/Refer Suspected Case | 33  (41.8%) | 17  (41.5%) | 16  (42.1%) |  | 20 (100%) | 13  (22%) |  | 14  (25.9%) | 19 (76%) |  |
| Diagnoses and Manages Stroke, n,(%) | | | | 0.7 |  | | **<0.001** |  | | **<0.001** |
| Diagnosis, treatment and follow up | 42  (53.2%) | 23  (56.1%) | 19  (50%) |  | 0  (0%) | 42  (71.2%) |  | 36  (66.7%) | 6  (24%) |  |
| Follow up Only | 3  (3.8%) | 2  (4.9%) | 1  (2.6%) |  | 0  (0%) | 3  (5.1%) |  | 3  (5.6%) | 0  (0%) |  |
| No Service/Refer Suspected Case | 34  (43%) | 16  (39%) | 18  (47.4%) |  | 20 (100%) | 14  (23.7%) |  | 15  (27.8%) | 19  (76%) |  |
| Provides all Four CVD Services, n,(%) | 29 (36.7%) | 16  (39%) | 13  (34.2%) | 0.7 | 0  (0%) | 29  (49.2%) | **<0.001** | 24 (44.4%) | 5  (20%) | **0.04** |

Supplementary Table 3: Model Interaction between Facility Ownership and LSHS Participation Status

| **Table S3: Model Interaction between Facility Ownership and LSHS Participation Status** | | | | |
| --- | --- | --- | --- | --- |
| Predictor | Estimate | Standard Error | t-value | p-value |
| Simple Linear Models with Interaction | | | | |
| Ownership x Public (R^2^ = 1.1%) | -5.644 | 4.132 | -1.366 | 0.18 |
| LSHS Status x Not Empaneled (R^2^ = 1.7%) | -5.855 | 3.823 | -1.532 | 0.13 |
| Model with Interaction (R^2^= 8.4%) | | | | |
| Ownership x Public | 0.07137 | 5.11439 | 0.014 | 0.1 |
| LSHS Status x Not Empaneled | -1.72840 | 4.41199 | -0.392 | 0.7 |
| Ownership x Public: LSHS Status x Not Empaneled | -18.01119 | 8.29084 | -2.172 | 0.03 |
